# Regulation of Fission Yeast Morphogenesis by PP2A Activator *pta2*

**DOI:** 10.1371/journal.pone.0032823

**Published:** 2012-03-05

**Authors:** Manuel Bernal, Maria Antonia Sanchez-Romero, Silvia Salas-Pino, Rafael R. Daga

**Affiliations:** Centro Andaluz de Biología del Desarrollo, Universidad Pablo de Olavide-Consejo Superior de Investigaciones Científicas, Junta de Andalucia, Sevilla, Spain; Cancer Research UK London Research Institute, United Kingdom

## Abstract

Cell polarization is key for the function of most eukaryotic cells, and regulates cell shape, migration and tissue architecture. Fission yeast, *Schizosaccharomyces pombe* cells are cylindrical and polarize cell growth to one or both cell tips dependent on the cell cycle stage. Whereas microtubule cytoskeleton contributes to the positioning of the growth sites by delivering polarity factors to the cell ends, the Cdc42 GTPase polarizes secretion via actin-dependent delivery and tethering of secretory vesicles to plasma membrane. How growth is restricted to cell tips and how re-initiation of tip growth is regulated in the cell cycle remains poorly understood. In this work we investigated the function of protein phosphatase type 2A (PP2A) in *S. pombe* morphogenesis by deleting the evolutionary conserved PTPA-type regulatory subunit that we named *pta2*. *pta2*-deleted cells showed morphological defects and altered growth pattern. Consistent with this, actin patches and active Cdc42 were mislocalized in the *pta2* deletion. These defects were additive to the lack of Cdc42-GAP Rga4. *pta2*Δ cells show upregulated Cdc42 activity and *pta2* interacts genetically with polarisome components Tea1, Tea4 and For3 leading to complete loss of cell polarity and rounded morphology. Thus, regulation of polarity by PP2A requires the polarisome and involves Pta2-dependent control of Cdc42 activity.

## Introduction

Cell polarity contributes to spatial organization of cells and is essential for morphogenesis, cell migration, asymmetric cell division and axon guidance [1], [2]. Cell polarization is usually triggered by internal or external cues and results in asymmetric organization of cellular cytoskeleton [3], [4]. The fission yeast *Schizosaccharomyces pombe*, amenable to a wide array of genetic and microscopy methods, is a convenient model for the study of cell polarity [5]. Fission yeast cells are rod-shaped, grow by tip extension and divide by medial fission, generating two daugther cells of equal size. Fission yeast polarization changes during cell cycle progression. These cells have a stereotyped pattern of growth where newly born cells after mitosis grow at one end, the “old end”, where growth occured prior to cell division. In G2, they initiate cell growth at the other end (the “new end”, generated by cell division). This transition from monopolar to bipolar growth is known as NETO (New End Take Off) [6], [7] and its molecular mechanisms are still poorly understood [8], [9].

As in other eukaryotic cells, fission yeast polarization depends on both the microtubule and actin cytoskeletons [10]–[12]. While actin polarization in fission yeast, similar to that in budding yeast, depends on scaffold dependent polarization and activation of Cdc42 GTPase and is essential for growth zone formation and cylindrical shape of fission yeast [13]–[15], an additional layer of microtubule-based control acts to determine the position of actin growth zones within the cell [10], [16], [17]. Microtubule-based delivery to the cell ends of polarity factors including the kelch repeat protein Tea1 and the SH3 domain containing Tea4/Wsh3p recruits the Pom1 kinase to cell tips and activates For3 formin responsible for the nucleation of actin cables, thus guiding growth zones [9], [16], [18]–[22]. For3 activity is controlled by an autoinhibitory interaction between its N-terminal inhibitory domain (DID) and the C-terminal autoregulatory domain (DAD) [23]. In fission yeast, full activation and proper localization of For3 is achieved by binding of Cdc42 to its N-terminus and of Bud6 to the C-terminal DAD domain, relieving the autoinhibitory conformation [24]. Cdc42 binding protein Pob1 is also required for For3 localization to the tips and facilitates Cdc42-mediated activation of For3 [25]. Actin cables nucleated by formins act as linear tracks along which exocytic vesicles can be transported by myosin-V towards the sites of actin nucleation [26]. Vesicles are then tethered by the exocyst complex to the cell membrane. Proper localization of the exocyst to the growth sites depends on both actin cables and Cdc42 [27], [28]. Therefore, Cdc42 is required for actin cable nucleation by For3 formin and also for the recruitment of the exocyst components to the growth zones [24], [27], [28]. The amount of active GTP-bound Cdc42 is regulated by guanine nucleotide exchange factors (GEFs) and GTPase activating proteins (GAPs) [29], [30]. Whereas the two known GEFs of Cdc42 in *S. pombe*, Scd1 and Gef1, localize to cell tips [4], [13], [31], the inhibitory GAP Rga4 localizes to the cell sides [32]. Rga4 is excluded from the tips by a mechanism that requires the Pom1 kinase and prevents ectopic growth away from the cell ends [31]–[34]. The NDR kinase Orb6 spatially restricts growth to cell tips by preventing Gef1 localization to the cell sides, thus providing further spatial control of cell growth [35]. Rga4 is also required for the normal localization of For3 and for the normal organization of the actin cytoskeleton [33]. In addition to the spatial control of Cdc42 activity, temporal regulation of Cdc42 GAPs and GEFs activity by Cdk1 might provide the cells with a mechanism that coordinates pattern of cell growth with cell cycle progression [36]–[38]. Once cell polarity has been established, feedback mechanisms involving Cdc42 regulation maintain this polarized state [1].

In this study, we investigate the role of protein phosphatase type 2A (PP2A) in the regulation of cell polarity and cell size in *S. pombe*. PP2A is one of the major serine/threonine phosphatases in eukaryotic cells [39]. It is involved in the regulation of a variety of cellular processes including cell cycle progression, cytokinesis, stress response and morphogenesis [39]–[41]. PP2A is a holoenzyme formed by a catalytic (C) subunit, a structural (A) subunit and a regulatory (B) subunit, which confers substrate specificity and regulates the subcellular localization of the PP2A complex. Different B and C subunits provide the cell with a set of distinct PP2A complexes with different substrate specificity [39], [42]. Proper assembly and activation of the PP2A holoenzyme also requires posttranslational modification of the catalytic subunit by a methyltransferase that catalyzes a reversible methylation of the C subunit at carboxyterminal leucine [43], [44]. In addition, binding of a phosphatase activator (PTPA) to both catalytic and scaffolding subunit is further required for the proper assembly and activity of PP2A complex [42], [45], [46]. The regulatory function of PTPAs is conserved through evolution from yeast to humans [46].

In this work, we characterize in *S. pombe* the function of a previously undescribed PP2A regulatory subunit of PTPA type that we named Pta2. Based on the *in vivo* Pta2 association with the PP2A complex and on the sequence analysis, we propose that *pta2* is the homologue of *S. cerevisiae* PTPA, *RRD2/YPA2* [46]–[49]. *pta2*Δ cells are cold-sensitive and have a number of morphogenetic phenotypes, including changes in cell shape and altered pattern of growth, as well as a difference in size at division between daughters. *pta2*Δ shows strong genetic interactions with Cdc42 regulators and with key polarity factors Tea1, Tea4 and For3. Our results demonstrate a role for *pta2* in PP2A dependent regulation of cell polarity and cell growth.

## Results and Discussion

### Identification of proteins associated with PP2A subunit paa1

**Figure 1:**
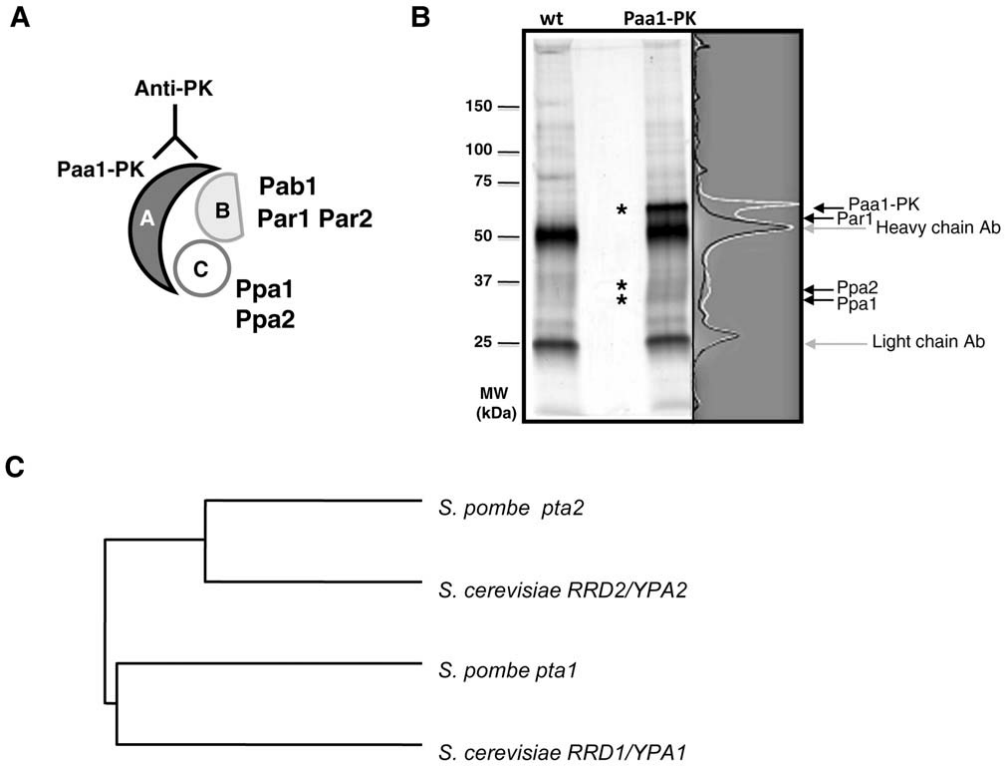
Identification of proteins associated with the PP2A complex subunit Paa1. **A.** PP2A complex composition. **B.** Proteins co-immunoprecipitated with PK-tagged PP2A structural subunit Paa1 were resolved by SDS-PAGE and detected by silver staining. Strain containing untagged Paa1 was used as control. **C.** Cladogram showing sequence similarity of two PTPA related genes present in the *S. pombe* and *S. cerevisiae* genomes.

In order to identify new regulators and/or substrates of the fission yeast PP2A complex we used co-immunoprecipitation followed by mass spectrometry analysis. The structural subunit of PP2A complex Paa1 [50] was tagged at the C-terminus with the PK epitope at the endogenous chromosomal locus and was expressed under the control of its native promoter (Figure 1A, scheme). The resulting protein fusion (Paa1-PK) was functional as judged by the absence of any observable phenotype (data not shown). Protein extracts from exponentially growing Paa1-PK expressing cells and untagged wild type cells as a control were subjected to immunoprecipitation with anti-PK tag antibody. Precipitated proteins were resolved by SDS polyacrylamide gel electrophoresis and detected using silver staining (Figure 1B). The immunoprecipitation experiment was repeated four times and consistently observed bands corresponding to Paa1-PK associated proteins were excised from the gel and identified using tandem mass spectrometry (Figure 1B and Table 1). Among Paa1 associated proteins we found the two catalytic subunits of PP2A, Ppa1 and Ppa2 [51]. We also found Par1, a regulatory B′ subunit of PP2A [52], [53], and two genes not characterized in *S. pombe*, SPBP8B7.08c and SPAC1782.05. SPBP8B7.08c is a homologue of Ppm1, a leucine carboxyl methyltransferase that in other organisms is required for PP2A complex assembly and activity [43], [44], [46], while SPAC1782.05 is a homologue of the PP2A phosphatase activator (PTPA) [48], [49] (Figure S1). A number of other putative Paa1-PK interacting proteins, some of which may be substrates of the PP2A complex, were also found (Figure S1). The two known B regulatory subunits of PP2A complex, the B′ type subunit Par2 and the B-type subunit Pab1, have not been identified in our pull down assay, presumably due to their low abundance [50]–[54]. We have selected PTPA homologue SPAC1782.05 for further study.

**Table 1:** Strain list.

Strain	Genotype	Source
RD 312	*h+ ade6-M210 ura4-D18 leu1-32*	Lab collection
RD 313	*h− ade6-M216 ura4-D18 leu1-32*	Lab collection
RD 1192	*h+ pta2::ura4+ade6-M216 ura4-D18 leu1-32*	This study
RD 1193	*h− pta2::ura4+ade6-M216 ura4-D18 leu1-32*	This study
RD 1439	*h+ paa1:pK ade6- leu1-32*	This study
RD 821	*h− pta2:GFP:kanMX6 ade6-M216 ura4-D18 leu1-32*	This study
RD 1349	*h pta2::ura4+tea1:GFP:kanMX6 ade6-M216 ura4-D18 leu1-32*	This study
RD 1292	*h pta2::ura4+for3:3GFP:ura4+ade6-M216 ura4-D18 leu1-32*	This study
RD 1298	*h pta2::ura4+rga4:GFP:kanMX6 ura4-D18 leu1-32*	This study
RD 1306	*h− pta2::ura4+CRIB:GFP:ura4+ura4-D18 leu1-32*	This study
RD 1304	*h− tea1:GFP:kanMX6 ade6-M216 ura4-D18 leu1-32*	Lab collection
RD 1139	*h− for3:3GFP-ura4+ade6-M216 leu1-32 ura4-D18*	Martin SG. *et al.*,2005
RD 1176	*h− rga4:GFP:kanMX6 ura4-D18 leu1-32*	Tatebe H. *et al.*, 2008
RD 1178	*h− CRIB:GFP-ura4+ura4-D18 leu1-32*	Tatebe H. *et al.*, 2008
RD 2064	*h− pta2::ura4+gef1::ura4+ade6-M216 ura4-D18 leu1-32*	This study
RD 1346	*h+ gef1::ura4+ade6-M216 ura4-D18 leu1-32*	Lab collection
RD 1266	*h pta2::ura4+tea4::kanMX6 ade6- ura4-D18 leu1-32*	This study
RD 1262	*h pta2::ura4+tea1::ura4+ade6-M216 ura4-D18*	This study
RD 1294	*h pta2::ura4+rga4::ura4+ura4-D18 leu1-32*	This study
RD 264	*h+ tea1::ura4+ura4-D18 ade6-M216*	Mata J. *et al.*, 1997
RD 1099	*h+ tea4::kanMX6 ade6- leu1-32 ura4-D18*	Martin SG. *et al.*,2005
RD 1177	*h− rga4::ura4+ura4-D18 lue1-32*	Tatebe H *et al.*, 2008
RD 1459	*h− for3::kanMX6 ade6- ura4-D18 leu1-32*	Lab collection
RD 1494	*h+ HA:cdc42 ade6-M16 leu1-32 ura4-D18*	Coll et all., 2005
RD 1505	*h pta2::ura4+HA:cdc42 ade6- ura4-D18 leu1-32*	This study

### *pta1* and *pta2* are homologous to *S. cerevisiae* phosphatase activators RRD1/YPA1 and RRD2/YPA2

The genome of *S. cerevisiae* contains two paralogous genes encoding PTPAs, RRD1/YPA1 and RRD2/YPA2 [48], [49]. In *S. pombe*, in addition to SPAC1782.05, we identified a second PTPA-related gene, SPAC4F10.04, by homology search. This gene shows 36% sequence identity to SPAC1782.05 at the amino acid level. Sequence comparison of *S. pombe* PTPAs with their homologues in *S. cerevisiae* revealed that SPAC1782.05 has higher amino acid sequence identity with RRD2/YPA2 whereas SPAC4F10.04 is closer to RRD1/YPA1 (Figure 1C and Figure S2). In *S. cerevisiae*, RRD2/YPA2 is required for the activity and P-Ser/P-Thr-specificity of the catalytic subunit of PP2A and associates *in vivo* with the two catalytic subunits of PP2A, RPH21 and RPH22, and with the structural subunit, TPD3 [46]. In contrast, RRD1/YPA1 is required for the activity of another PP2A-like phosphatase, SIT4, with which it interacts physically and genetically [46], [49], [55]. Thus, both the higher sequence identity between *S. pombe* SPAC1782.05 and *S. cerevisiae* RRD2/YPA2 and the physical association of SPAC1782.05 with PP2A complex found in our co-immunoprecipitation experiments suggest that SPAC1782.05 is homologous to *S. cerevisiae* RRD2/YPA2. Thus, we named this gene *pta2*.

### Subcellular localization of Pta2

**Figure 2:**
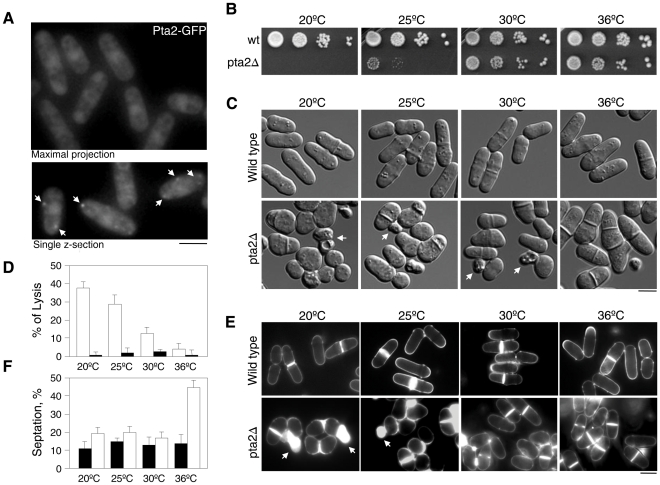
Subcellular localization of Pta2 and phenotypic characterization of *pta2* Δ. **A.** Subcellular localization of Pta2-GFP expressed from its endogenous promoter. Top panel, maximum Z-projection, bottom panel, single z-section. Arrows point to cytoplasmic foci of Pta2-GFP. **B.** Growth assay of serial dilutions of wild type and *pta2*Δ cells at the indicated temperatures. **C.** Differential interference contrast (DIC) images of wild type and *pta2*Δ cells grown at the indicated temperatures. Scale bar, 5 µm. **D.** Bar graph showing percentage of cells displaying a lytic phenotype in wild type and *pta2*Δ strains. **E.** Images of wild type and *pta2*Δ cells stained with calcofluor at the indicated temperatures. **F.** Bar graph showing percentage of septated cells of wild type and *pta2*Δ strains.

To understand *pta2* function within the *S. pombe* cell, we first analyzed its subcellular localization by tagging the endogenous *pta2* locus with the *green fluorescent protein* (GFP). The fusion was expressed under its own promoter and was functional as judged by the phenotype of *pta2-GFP* cells that was indistinguishable from wild type. Pta2-GFP localized to both the cytoplasm and the nucleus, showing some nuclear enrichment (Figure 2A, asterisks). Similar results were obtained when *pta2-GFP* was expressed from a multicopy plasmid controlled by the *nmt41* promoter (data not shown). Pta2-GFP also localized at discrete motile foci at the cytoplasm (Figure 2A, arrows, and data not shown) that could possibly correspond to vesicles. Diffuse localization of Pta2-GFP was similar to localization of other components of PP2A complexes in fission yeast, the main catalytic subunit Ppa2, the B-type subunit Pab1 and the B′-type subunits Par1 and Par2, though the latter two proteins seem to be excluded from the nucleus [8], [52], [54].

### Phenotypic characterization of *pta2*Δ

To further characterize the role of *pta2* in *S. pombe* we generated a deletion of this gene by replacing the entire open reading frame with the *ura4* marker. Deletion of *pta2* was viable but displayed distinct phenotypic alterations compared to the wild type *S. pombe*. Similar to the lethality of *rrd1*Δ*rrd2*Δ strain lacking the two PTPA proteins in *S. cerevisiae* [48], a double deletion of the two PTPA related genes in *S. pombe pta1*Δ*pta2*Δ was also lethal (data not shown). *pta2*Δ cells were slow growing and cold-sensitive (Figure 2B, and data not shown) and showed morphological defects ranging from a pear-like phenotype with cells displaying different width at cell tips, to an almost round morphology which was more penetrant at lower temperatures (Figure 2C). We also observed high frequency of cell lysis that was also more pronounced at lower temperatures (38% of cells lysing at 20°C, n = 193), (Figure 2D). The lytic phenotype was not suppressed by sorbitol (Figure S3) and cell death occurred mostly during cytokinesis (data not shown). We also found a significant increase in the number of septated cells in *pta2*Δ at 36°C, comprising 44% (n = 255 cells) of the population compared to 14% n = 221 cells) in the wild type strain in the same conditions (Figure 2E, F), suggesting defects in the late stages of cytokinesis at 36°C similar to those resulting from the loss of function of PP2A B′-type *par1* and *par2* and B-type *pab1* [50], [52]–[54]. Thus, the phenotypic analysis of *pta2*Δ strain suggests that PP2A-Pta2 has a role in cell morphogenesis and cytokinesis.

### Partial loss of cell polarity in *pta2Δ* cells

**Figure 3:**
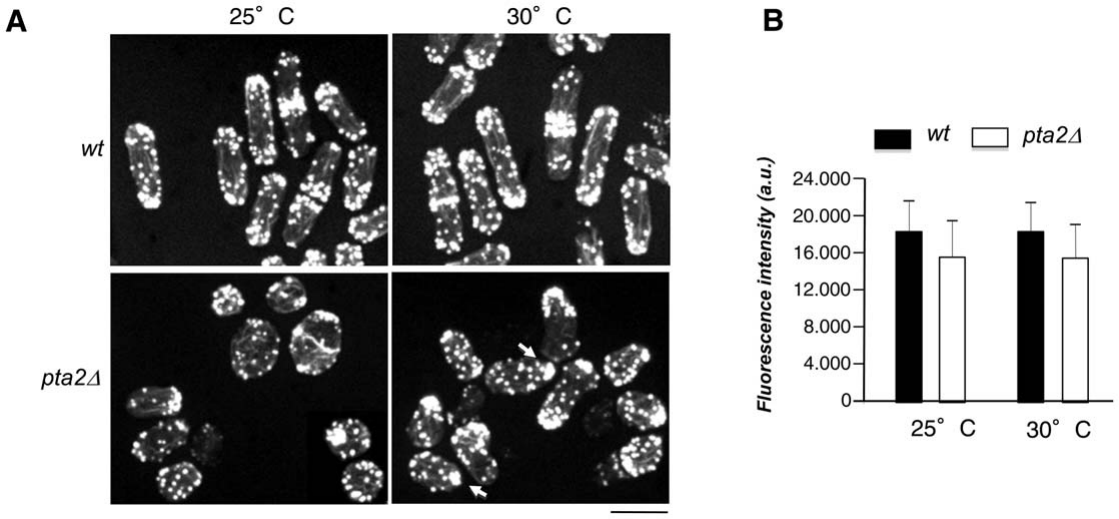
*pta2* is required for polarization of actin cytoskeleton. **A.** F-actin staining using Alexa fluor 488-conjugated phalloidin of wild type and *pta2*Δ cells at the indicated temperatures. Images were recorded in multiple focal planes, maximum projections are shown. Scale bar, 5 µm. **B.** Bar graph showing actin cable intensity in wild type and *pta2*Δ cells at the indicated temperatures.

**Figure 4:**
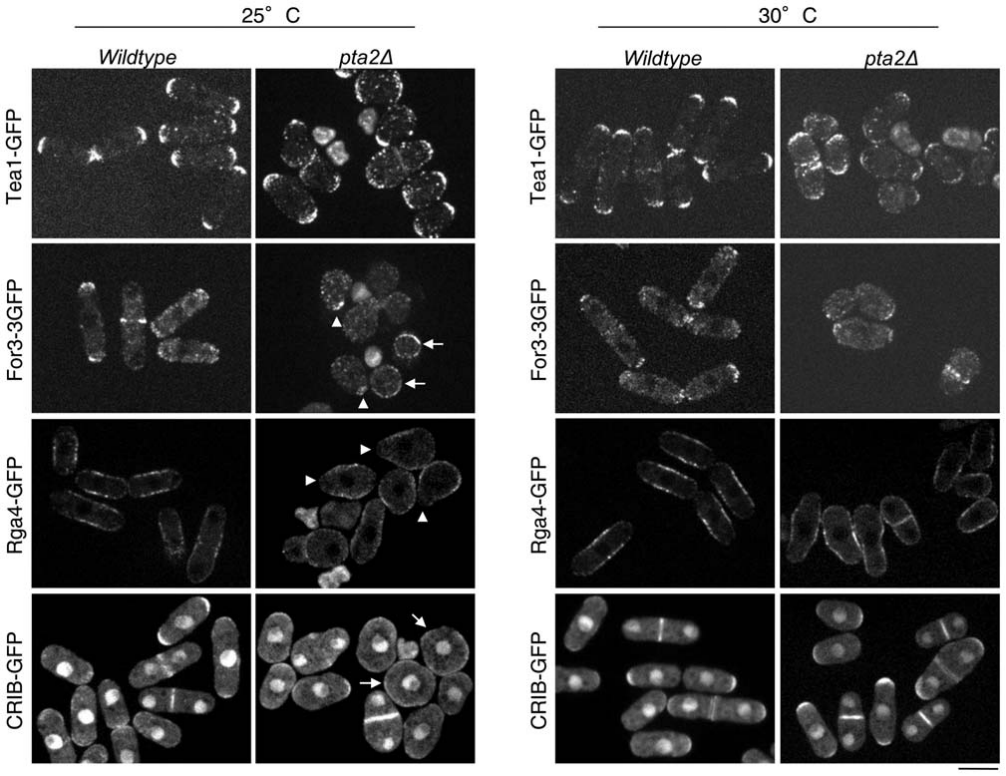
Subcellular localization of key polarity markers in wild type and *pta2* Δ cells. Fluorescence microscopy images of indicated GFP-fusions of polarity markers in the wild type and *pta2*Δ strains were grown at 30°C. Tea1-GFP, For3-GFP Rga4-GFP and CRIB-GFP images were recorded in multiple focal planes, maximum projections are shown for Tea1-GFP, For3-3GFP and CRIB-GFP and a single medial section for Rga4-GFP. Arrows indicate unpolarized cells. Arrowheads show hyperpolarized cells with one pointed end. Scale bar, 5 µm.

To better understand the origin of the morphological defects observed in the *pta2*Δ cells, we examined actin and microtubule (MT) cytoskeletons in *pta2*Δ cells using fluorescent phalloidin labeling and anti-tubulin immunofluorescence, respectively. MT cytoskeleton in *pta2*Δ cells was apparently normal with bundles extending across the whole cell length and reaching the cell tips (Figure S4). In pear-like and in rounded *pta2*Δ cells MT bundles were more disorganized, possibly as a consequence of altered cell shape (Figure S4). Actin cytoskeleton organization in *pta2*Δ strain was very different from the wild type interphase *S. pombe* cells. Actin patches polarization at the cell tips seen in the wild type (Figure 3A) [56] was frequently defective in *pta2*Δ cells that displayed a range of patterns from mostly disorganized actin patches at 25°C, to a more polarized wild type phenotype or even hyperpolarized cells with a single cluster of patches at a pointed end (Figure 3A, 30°C, arrows). The number and intensity of actin cables in *pta2*Δ cells were similar to those in wild type cells, but their distribution was altered in more rounded cells (Figure 3A, B). Using fluorescence microscopy, we further analyzed localization of GFP fusions of key regulators of cell polarity in *pta2*Δ cells, the cell end marker Tea1 and the formin For3. We also examined the spatial distribution of Cdc42 GAP Rga4 and used GFP-tagged CRIB reporter to localize active GTP-Cdc42 [32], [57]. At optimum growth temperature (30°C) *pta2*Δ cells cells showed polarized Tea1-GFP and For3-3GFP localization, Rga4 was excluded from cell tips and CRIB-GFP localized to either one or both cell tips (Figure 4). At lower temperatures, however, *pta2*Δ cells showed unpolarized For3-3GFP, Rga4-GFP and CRIB-GFP localization spread across the whole cellular surface (Figure 4). Surprisingly, in spite of the unpolarized localization of these polarity markers in most cells, we also found hyperpolarized *pta2*Δ cells containing a bright focus of actin, Tea1-GFP and For3-3GFP at one single pointed tip (Figure 4, arrowheads). This heterogeneity of phenotypes could be explained either by a different cell cycle stage of hyperpolarized cells [58] or by the ability of unpolarized *pta2*Δ cells to form unstable hyperpolarized growth zones.

Thus, PP2A-*pta2*Δ has defects in F-actin organization, in localization of For3 and active Cdc42, likely causing their altered morphology. We conclude that PP2A regulation by *pta*2 controls assembly and/or maintenance of growth zones in *S. pombe*.

### PP2A-Pta2 regulates growth zone positioning during the cell cycle

**Figure 5:**
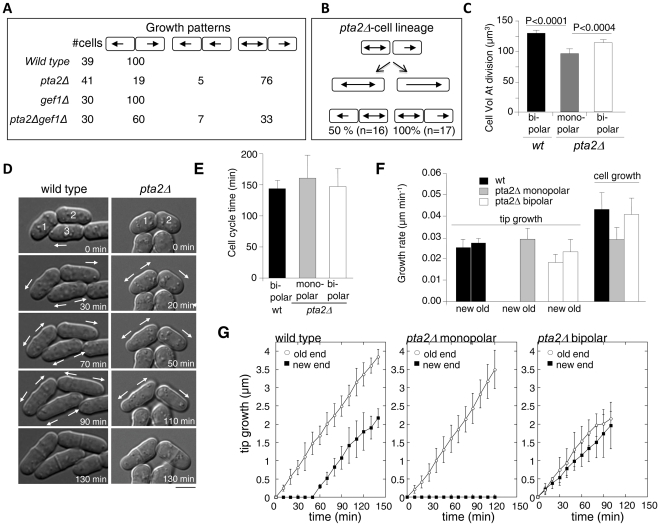
*pta2* >Δ cells show a change in growth pattern. **A.** Schematic representation of growth patterns after division and the percentage of cells in indicated strains displaying each pattern. **B.** Schematic representation of cell lineages of *pta2*Δ cells. **C.** Bar graph showing cell size at division in wild type and in monopolar and bipolar daughter cells of *pta2*Δ strain grown at 30°C. **D.** DIC time-lapse images of wild type and *pta2*Δ cells, respectively. Arrows indicate direction of cell growth. Images were recorded in 10 minute intervals at 30°C. **E.** Bar graph showing cell cycle timing from septation to the following mitosis. **F,G.** Rates of tip growth and cell growth in wild type, as well as in monopolar and bipolar *pta2*Δ daughter cells.

To determine whether the observed changes in the actin cytoskeleton in *pta2*Δ cells influence the ability of these cells to form new growth zones after cell division, we analyzed their growth patterns by DIC time-lapse microscopy at 30°C. At this temperature most cells remain polarized (Figure 2C, E). Our analysis revealed that, in contrast to the wild type cells, where all cells initiate the cell cycle growing in a monopolar manner (Figure 5A, D and Video S1) [7], in *pta2*Δ the two daughter cells differed from each other in their growth patterns after separation. After most divisions (76% n = 41 cells), one daughter grew in a monopolar manner using its old tip, and the other grew in a bipolar manner from the beginning of the cell cycle (Figure 5A, D and Video S2). In the remaining 24% of divisions, cells grew either at both old ends or at one new and one old end (Figure 5A). Lineage analysis of *pta2*Δ cells revealed that monopolar and bipolar cells were both able to generate one monopolar and one bipolar daughter after division (Figure 5B). All bipolar daughter cells (n = 37) contained the scar from the previous division, similar to what has been reported for *for3*Δ mutant [20], though how the scar contributes to cell polarity is not clear.

Surprisingly, monopolar and bipolar daughters enter mitosis at different sizes. Bipolar cells always divided at volumes around 15% larger than their monopolar sisters (115±10 µm^3^, n = 21 bipolar cells versus 97±14 µm^3^, n = 14 monopolar cells, Figure 5C).

This is the first example, to our knowledge, where in the fission yeast two daughter cells enter mitosis at different sizes after a symmetric division in a previous mitosis. Analysis of growth rates and cell division timing in the two daughters has revealed that growth rates were very different in monopolar and bipolar cells (0.029±0.005 µm min-1 bipolar n = 10 cells versus 0.041±0.003 n = 10 cells monopolar cells, p<0.0001) (Figure 5F, G), whereas the timing of mitotic entry was similar in the two daughter cells (148±29 min bipolar n = 10 cells versus 161±38 min monopolar cells n = 21 cells p<0.5) (Figure 5E). We interpret these observations as evidence that G2/M cell size control checkpoint that coordinates growth and division in *S. pombe* [59], [60] is unable to detect and/or compensate for the difference in growth rates between the two daughters in *pta2*Δ background. Thus, lack of PP2A function in *S. pombe*, in agreement with previously reported results [51], is negatively regulating cell size at division. Some of this regulation may be due to the ability of PP2A to regulate growth pattern and growth rates.

The premature mitotic entry in *pta2*Δ cells (Figure 5C, D) resembles the reported phenotypes of both the catalytic and regulatory B-type subunits of PP2A phosphatase [61], [62], consistent with synthetic lethality that we observe in *pta2*Δ*wee1-50* cells (Figure S5) and with a reported function of PP2A in the regulation of phosphorylation state and activity of Cdc25 that has been described in *S. cerevisiae* and *X. laevis* [63], [64].

It is possible that dependence of division size on the growth pattern could be a consequence of advanced mitosis in the *pta2* mutant. Deregulation of the normal mitotic control could potentially sensitize cell size checkpoint in *pta2*Δ to the asymmetry between the daughters.

Thus, PP2A-Pta2 regulates activation of growth at the new ends formed by cell division. It seems to promote identical growth pattern in the wild type daughters by preventing premature new end growth in one daughter cell. Since cells lacking *for3* formin display similar asymmetric growth patterns in the two daughters [20], it is possible that changes in Cdc42 dependent actin cable nucleation by formin are responsible for the asymmetric growth of daughters in *pta2*Δ.

Consistent with this hypothesis, deletion of *cdc42* activator *gef1* in *pta2*Δ cells significantly decreased the number of divisions producing asymmetric bipolar/monopolar growth of daughter cells, from 76% to 33% (Figure 5A). A recent report suggesting similar changes in growth pattern upon ectopic activation of Cdc42 caused by *rga4* deletion is providing additional support to our model [33]. In contrast, mutants in polarity factors (*tea1*, *tea4* and *pom1*) that also alter growth patterns but do not interfere with Cdc42 regulation only generate monopolar cells [65].

### Genetic interaction between Pta2 and cell polarity determinants

**Figure 6:**
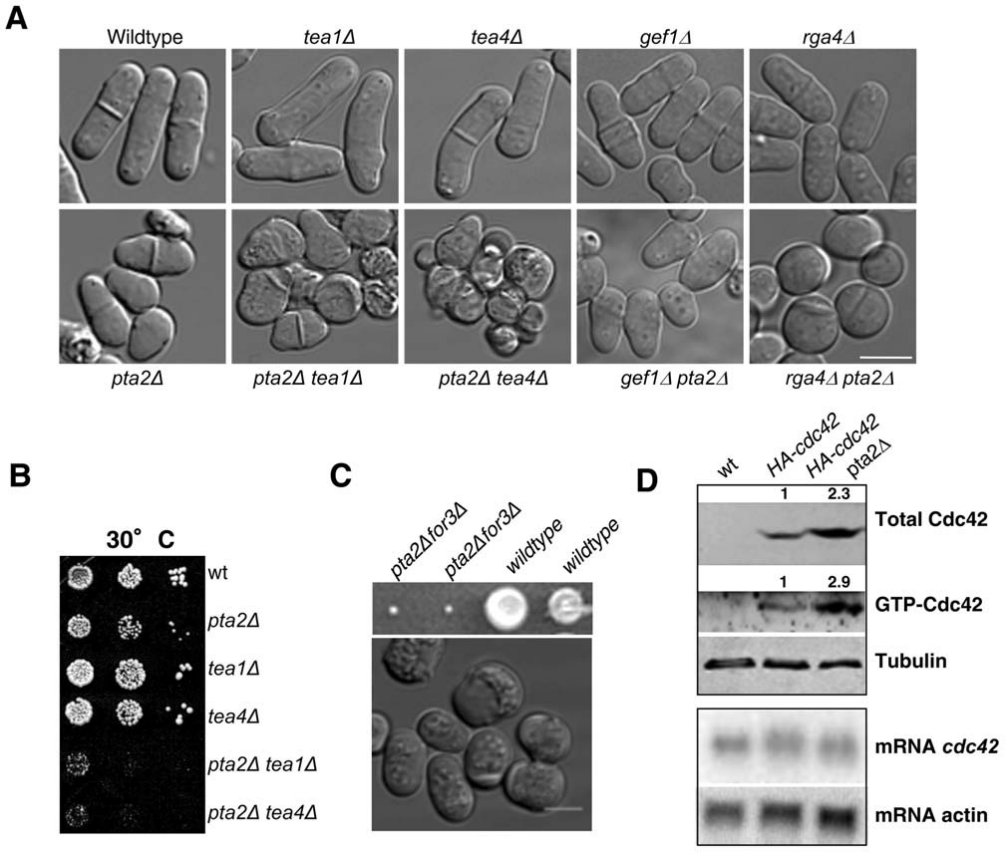
Cell polarization defects of *pta2*Δ cells are additive to deletions of key polarity regulators. **A.** DIC images of the indicated single and double mutants grown at 30°C. **B.** Growth assay of indicated single and double mutants at 30°C. **C.** Top, a tetrad showing the four meiotic products of a cross between *pta2*Δ and *for3*Δ. Bottom, a representative DIC image of *pta2*Δ*for3*Δ double mutant from a microcolony **D.** Top, western blot of total cell extracts prepared from *HA-cdc42* and *pta2*Δ* HA-cdc42* strains. GTP-bound Cdc42 affinity purified using bacterially expressed GST-CRIB. Western blots were probed with either anti-HA antibody to detect Cdc42 or anti-tubulin antibody as loading control. Numbers above the gels show relative amounts of protein in the bands. Bottom, northern blot showing mRNA levels of *cdc42* in a wild type and *pta2*Δ backgrounds.

In *S. pombe*, growth site selection is achieved by the delivery of polarity factors including Tea1 and Tea4 to cell tips and subsequent activation of actin polymerization via formin For3 [9]. Exponentially growing *tea1*Δ and *tea4*Δ cells are cylindrical, slightly bent and grow in a monopolar manner with F-actin patches located at the single growing tip [16], [22], [66] (Figure 6A). To check whether defects of *pta2*Δ in the establishment of cell polarity were additive or epistatic to deletion of *tea1* and *tea4* we generated double mutants of these genes and *pta2*Δ. Deletion of *pta2* in the *tea1*Δ or *tea4*Δ backgrounds resulted in an almost complete loss of cell polarity and a drastic impairment of cell growth (Figure 6A, B). This additive phenotype is similar to what is seen when actin cables are compromised in the Tea1 deletion [21]. To check whether *pta2*Δ and *for3*Δ were epistatic for their cell morphology phenotypes, we produced double mutant *pta2*Δ*for3*Δ. Spores of *pta2*Δ*for3*Δ germinated and slowly proliferated, forming microcolonies (Figure 6C). Microscopic observation of these cells showed that most of them eventually became unpolarized (Figure 6C). The strong negative interaction between *pta2* and *for3* genes is reminiscent of complete loss of cell polarity in double mutants of *for3* with exocyst components [27], [28], raising the possibility that PP2A might be regulating exocyst function.

Thus, cell polarization defects of *pta2*Δ cells are additive to defects in microtubule and actin-dependent mechanisms of cell polarization.

### PP2A-Pta2 regulates levels and activity of Cdc42

Cdc42 is a key regulator of polarity and *cdc42* mutants in fission yeast exhibit abnormal morphology [14], [29]. At the molecular level, Cdc42 is required for actin cable formation and also for tethering exocytic vesicles to the sites of growth [27], [28]. In addition to the described genetic interactions of *pta2*Δ with both MT- and actin-dependent mechanisms of cell polarization, we also found a negative genetic interaction between *pta2*Δ and the deletion of the exocyst component Exo70 (data not shown). Thus, we tested whether changes in cell morphology and growth site selection seen in *pta2*Δ cells can involve changes in the Cdc42 activity. To this end, we analyzed genetic interactions between *pta2*Δ and two regulators of the Cdc42 pathway, *gef1*Δ (Cdc42 GEF) and *rga4*Δ (Cdc42 GAP) [4], [32], [33], [67]. Apart from partial suppression of daughter cell asymmetry (Figure 5A), no significant changes were seen in *gef1*Δ*pta2*Δ compared to *pta2*Δ cells (Figure 6A). In contrast, morphological defects of *pta2*Δ cells were exacerbated in *pta2*Δ*rga4*Δ double mutant, resulting in a complete loss of polarity (Figure 6A). Neither *gef1*Δ nor *rga4*Δ suppressed cold-sensitivity of *pta2*Δ (data not shown). Since Gef1 is an activator of Cdc42 and Rga4 is an inhibitor, the phenotypes of double mutants are consistent with higher Cdc42 activity in the *pta2*Δ.

To directly measure the amounts of Cdc42 in *pta2*Δ cells we assayed total levels of Cdc42 by western blot using HA-Cdc42 and levels of active Cdc42 by a pull down assay using bacterially expressed PBD-domain fused to GST, which binds GTP-bound Cdc42, as a bait [68]. The levels of active Cdc42 were increased in *pta2*-deleted strain, consistent with the above genetic interaction data (Figure 6D). Surprisingly, the total amounts of Cdc42 were also elevated (Figure 6D). To check whether the changes in total Cdc42 protein levels observed in *pta2*Δ cells were due to transcriptional upregulation or to changes in protein synthesis or degradation, we analyzed by northern blot Cdc42 mRNA levels in the *pta2* deletion. We found no changes in *cdc42* mRNA levels in the *pta2*Δ background relative to those in wild type (Figure 6D), suggesting that PP2A might be directly or indirectly regulating Cdc42 translation or turnover.

The two distinct morphogenetic phenotypes seen in *pta2*Δ cells, altered growth pattern [33], [69] and loss of cell polarity [14], [29] are both consistent with the role of Pta2 in downregulating Cdc42.

In summary, *pta2*Δ cells have several phenotypes highlighting the involvement of PP2A in the regulation of morphogenesis and the cell cycle. Alterations in cell polarity and cell shape in *pta2*Δ cells as well as the altered growth pattern suggest a role for PP2A in re-organizing actin machinery after division and in maintaining polarized growth zones. Morphological defects of *pta2*Δ are additive to defects in MT-dependent mechanism of cell polarization (*pta2*Δ*tea1*Δ or *pta2*Δ*tea4*Δ) and also to the absence of actin cables (*pta2*Δfor3Δ) resulting in both cases in the loss of cell polarity and isotropic growth. Interestingly, whereas the altered growth pattern of *pta2*Δ cells was partially suppressed by a deletion of Cdc42 GEF, Gef1, the morphological defects of *pta2*Δ cells were additive to the deletion of Cdc42-GAP Rga4. We found that PP2A-Pta2 regulates the stability or recycling of Cdc42 GTPase as *pta2*Δ cells have higher levels and activity of Cdc42. Although lack of function of *rga4* does not have a big impact on total levels of GTP-bound Cdc42 [33] *rga4*Δ cells lose spatial control of this GTPase since Rga4 localizes to the sides of wild type cylindrical cells of *S. pombe* and is excluded from the cell tips, thus allowing cell growth to occur only at the cell tips [32]. It has been proposed that Rga4 negatively regulates cell growth at the site of division by preventing premature activation of growth at the new end [33]. It is possible that higher levels of active Cdc42 caused by lack PP2A function have the same consequence that lack of Rga4, giving rise to the same alteration of the growth pattern. Taken together, our results suggest that *pta2*Δ polarity defects might be, at least in part, due to increased levels of active Cdc42.

Thus, the function of PP2A in cell polarity involves Pta2-dependent down regulation of the Cdc42 GTPase, which cooperates with Cdc42 spatial control by microtubules and actin in the regulation of cell polarity.

## Materials and Methods

### Yeast strains and methods

Media, growth and maintenance of strains were as described in [70]. Cells were grown at 20°C, 25°C, 30°C and 36°C in liquid YES medium and cell morphology was analyzed by DIC microscopy. Septa and patterns of cell growth were visualized using 35 µg/ml calcofluor staining (fluorescent brightener; Sigma). Nuclei were visualized with 0.2 µg/ml DAPI (Sigma) or 100 µg/ml propidium iodide (Sigma) staining.

### Co-immunoprecipitation assay

Overnight cultures of PK-tagged Paa-1 (Paa1-PK) and untagged wild type strains were grown at 30°C to OD_600_ 0.35. 100 mls of culture were harvested by centrifugation (5 min, 4000 r.p.m., 4°C) and re-suspended in 4 ml of cold IP buffer (25 mM Tris pH 7.5, 0.1% Nonidet P-40, 150 mM NaCl). Samples were then centrifuged, resuspended in 0.1 ml of cold IP buffer containing protease inhibitor cocktail (Roche), transferred to eppendorf tubes containing glass beads and cells were then broken in the Fast-Prep bead-beater for 4 seconds at 4°C. Supernatant was centrifuged at 14,000 r.p.m. at 0°C for 10 minutes to remove cellular debris.

### Isolation and analysis of protein complexes

Cleared lysates containing 1 mg of total protein were mixed with 1.5 mg of Protein A Dynabeads (Invitrogen) cross-linked to mouse anti-PK antibody (kindly provided by Dr. I. Hagan, Paterson Institute, Manchester, UK) and incubated at 4°C on a rotating wheel for 60 minutes. Dynabeads were collected with a magnet and washed three times with 0.2 ml of cold IP buffer in 2 ml unsiliconized tubes (Eppendorf). IP buffer was removed from the beads and antigen-antibody-Dynabeads complex was dissolved in SDS-PAGE loading buffer containing 10 mM *tris* (2-carboxymethyl)-HCl (Sigma) at 95°C for 5 min. Samples were resolved by SDS-PAGE in 4–12% gradient gels (BioRad), and visualized by Silver Stain Kit (Pierce). To identify proteins present in distinct bands in the *paa1*-PK strain relative to untagged control, 1 mm gel slices were excised, destained and digested with trypsin (Promega) for 4 h at 37°C. Peptides from each slice were analyzed on a Thermo-Finnigan FT-ICR mass spectrometer using a NanoMate chip-based electrospray system operated by the University of Birmingham Functional Genomics and Proteomics Units.

### Generation of *pta2* gene deletion

*pta2* was deleted by the one-step gene replacement method using a PCR fragment containing the ura4 marker flanked by 728 bp upstream of the open reading frame of *pta2* gene and by 415 bp that correspond to a downstream region of *pta2* open reading frame. This construct was prepared in three steps: 1. The upstream region of *pta2* was amplified using the primers: 5′ GACTAGTCGGCTGCAAGCATTTATGAGTCTC 3′ and 5′ GGAATTCCGGCATAACTTGATCGGTTAAGAC 3′, the PCR product was digested with Spe1 and EcoRI and cloned into pBIISK. 2. The *ura4* cassette was digested with EcoRI and XhoI and cloned into the plasmid containing the upstream region of *pta2*. 3. The downstream region of *pta*2 gene was amplified with primers: 5′ GGCATCAAGATACCAAGTGCGATC 3′ and 5′ GGGGTACCCCATGTATCTGCTTCTAGCAGC 3′, digested with KpnI and XhoI and cloned into pBIISK containing the upstream region of *pta2* and the *ura4* cassette. Finally, the whole construct containing 2827 bp was amplified using the primers 5′ GGCTGCAAGCATTTATGAGTCTC 3′ and 5′ AT GTATCTGCTTCTAGCAGC 3′. The resulting PCR fragment was transformed into a diploid strain. Ura4 positive colonies were selected and gene replacement was confirmed by PCR and southern blot.

### Northern blotting

The total RNA from wild type and *pta2*Δ strains was extracted from overnight cultures using the QIAGEN RNeasy Mini kit. A 20 µg sample of RNA was run on a gel, transferred to a nitrocellulose membrane and hybridized with either 449 bp fragment of *cdc42* gene (position 711–1160 ORF) and a 449 bp fragment of *actin* gene (position 591–1040 ORF). The autoradiographic detection of the bands was performed using a Molecular Dynamics PhosphoImager.

### Western blotting and GTP-Cdc42 pull-down assay

To analyze levels of total and GTP-bound Cdc42 protein, chromosomally tagged *HA-cdc42* and *HA-cdc42 pta2*Δ strains (kindly provided by Dr P. Perez) were grown at 30°C in EMM to mid-exponential phase (0.35 OD_600_). Protein extracts were prepared in the buffer B (50 mM Tris-HCl pH 7.6, 20 mM NaCl, 0.1 mM DTT, 0.5% NP-40, 2 mM MgCl, 10% glycerol) containing protease inhibitor cocktail (Roche). Total amount of Cdc42 was determined by Western blotting using the anti-HA antibody (Sigma) and anti-mouse IgG-peroxidase (Sigma) secondary antibody. GTP-bound Cdc42 proteins were purified by binding to GST-PBD as described in [69].

### Cytoskeleton staining

Actin staining was performed as described in [71], using AlexaFluor 488-phalloidin (Molecular Probes).

For anti-tubulin immunofluorescence, cells were fixed in methanol at −80°C and further processed as described in [72]. Primary antibodies were anti-tubulin (TAT-1, used at 1∶80 dilution) followed by Alexa 488 goat anti-mouse secondary antibody (Molecular Probes).

### Microscopy

Live cell imaging was performed at 20–36°C using spinning disc confocal (Olympus IX81, Roper Scientific) and Delta-vision wide-field (Olympus IX71, Applied precision) microscope systems. For growth pattern analysis, cells from over night cultures were mounted on agar pads as described in [73]. Live-cell imaging was done at the constant temperature of 30°C. Data were acquired using the 100× objective (UPLANSAPO, NA 1.4) recording 15 z-sections with 0.3 µm spacing.

### Data analysis

Cell length and actin cables intensity were measured from a single z-section using ImageJ software (NIH, Bethesda, MD). To determine statistical significance of cell growth and cell volume changes we used the t-test. For cell volume measurements, DIC images were taken. Cell width and cell length were measured using ImageJ software and cell volumes were calculated by approximating cell shape as a cylinder capped by two hemispheres.

## Supporting Information

Figure S1 **List of Paa1 interacting proteins obtained in a Co-Immunoprecipitation assay.** 1. List of known and predicted subunits and regulators of PP2A complex 2. List of potential PP2A substrates and/or interacting proteins.
(TIF)

Figure S2 **Clustal analysis of** ***S. pombe*** **and** ***S. cerevisiae*** **phosphatase activators.** Protein sequence alignments of *S. pombe pta1* and *pta2* and *S. cerevisiae* RRD1/YPA1 and RRD2/YPA2 were performed using Clustal. Red boxes show three highly conserved domains that are shared by all PTPAs. The consensus sequence is shown in black.
(TIF)

Figure S3 **The cold sensitive phenotype of** ***pta2*** **Δ cells is not suppressed by 1 M sorbitol.** **A.** Growth assay of serial dilutions of wild type and *pta2*Δ cells at the indicated temperatures in YES agar containing 1 M sorbitol. **B.** Differential interference contrast (DIC) images of wild type and *pta2*Δ cells grown at the indicated temperatures. in YES medium containing 1 M sorbitol. Scale bar, 5 µm.
(TIF)

Figure S4 **Microtubule cytoskeleton in wild type and** ***pta2*** **Δ cells.** Wild type and *pta2*Δ cells were grown at either 25°C or 30°C and then fixed with methanol and incubated with anti-tubulin (TAT1) primary antibody and anti-mouse Alexa fluor 488 secondary antibody. Images were taken in multiple focal planes, maximum projections are shown. DAPI staining is shown in bottom panels. Scale bar, 5 µm.
(TIF)

Figure S5 **Synthetic lethality of** ***pta2*** **Δ** ***wee1-50*** **strain at 36°C.** **A.** DIC images of the indicated strains after 6 hours incubation at 36°C. **B.** The *pta2*Δ*wee1-50* strain was grown at 25°C and then shifted to 36°C (at time 0 hrs). Images were taken every 5 minutes at a constant temperature of 36°C, representative time points are shown. Scale bar, 5 µm.
(TIF)

Video S1 DIC time-lapse images taken at 10 minute intervals showing growth pattern of wild type cells at 30°C. Total time, 4 hours and 20 minutes
(MOV)

Video S2 DIC time-lapse images taken at 10 minute intervals showing growth pattern of *pta2*Δ cells at 30°C. Total time, 4 hours and 10 minutes.
(MOV)
