# Supplementary figures and images for: Regulation of Fission Yeast Morphogenesis by PP2A Activator pta2
Source: PLoS One. 2012 Mar 5;7(3):e32823. doi: 10.1371/journal.pone.0032823 (PMC3293916; doi:10.1371/journal.pone.0032823)

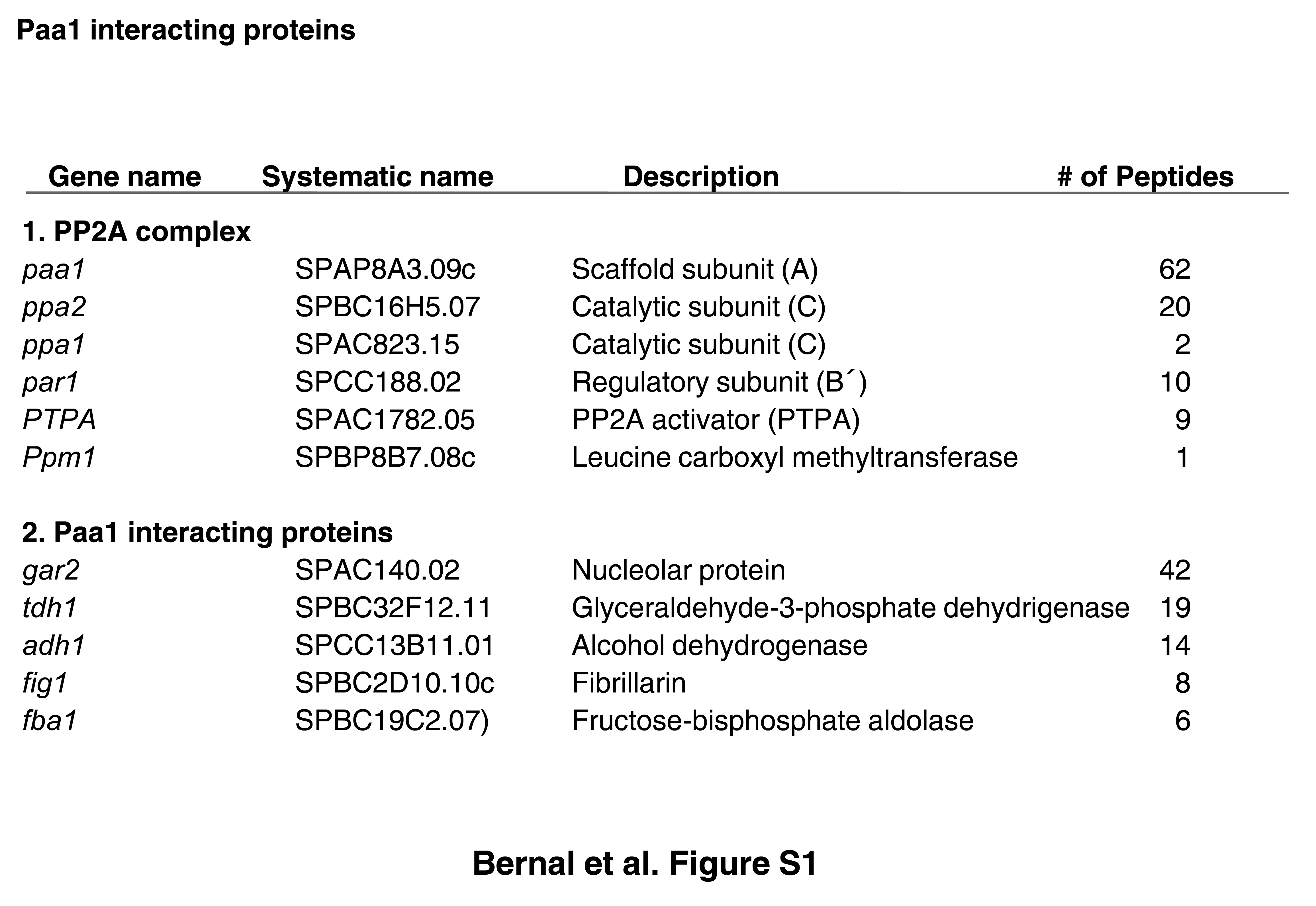

Supplement: Figure S1 — List of Paa1 interacting proteins obtained in a Co-Immunoprecipitation assay. 1. List of known and predicted subunits and regulators of PP2A complex 2. List of potential PP2A substrates and/or interacting proteins. (TIF) [file pone.0032823.s001.tif]

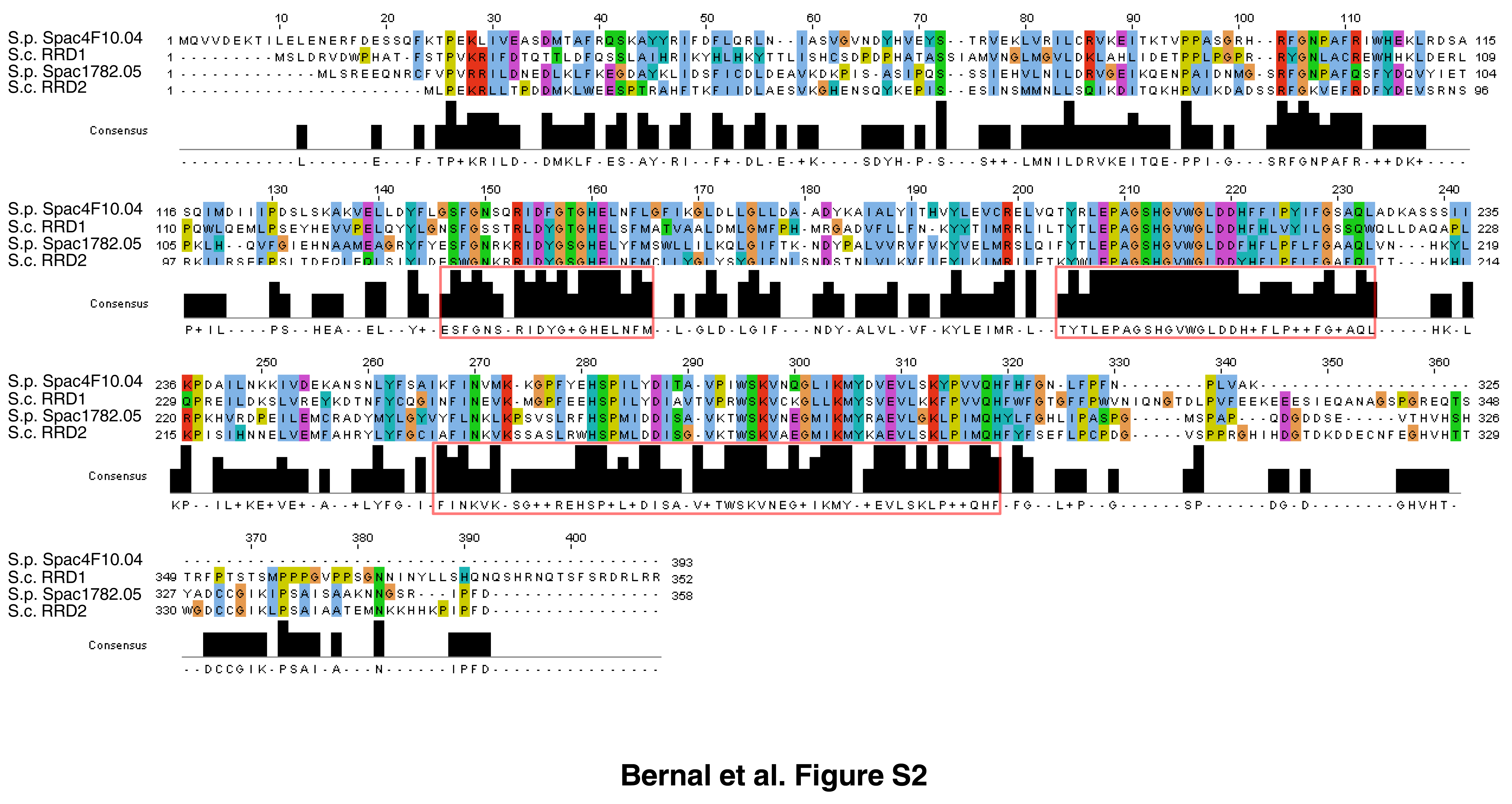

Supplement: Figure S2 — Clustal analysis of S. pombe and S. cerevisiae phosphatase activators. Protein sequence alignments of S. pombe pta1 and pta2 and S. cerevisiae RRD1/YPA1 and RRD2/YPA2 were performed using Clustal. Red boxes show three highly conserved domains that are shared by all PTPAs. The consensus sequence is shown in black. (TIF) [file pone.0032823.s002.tif]

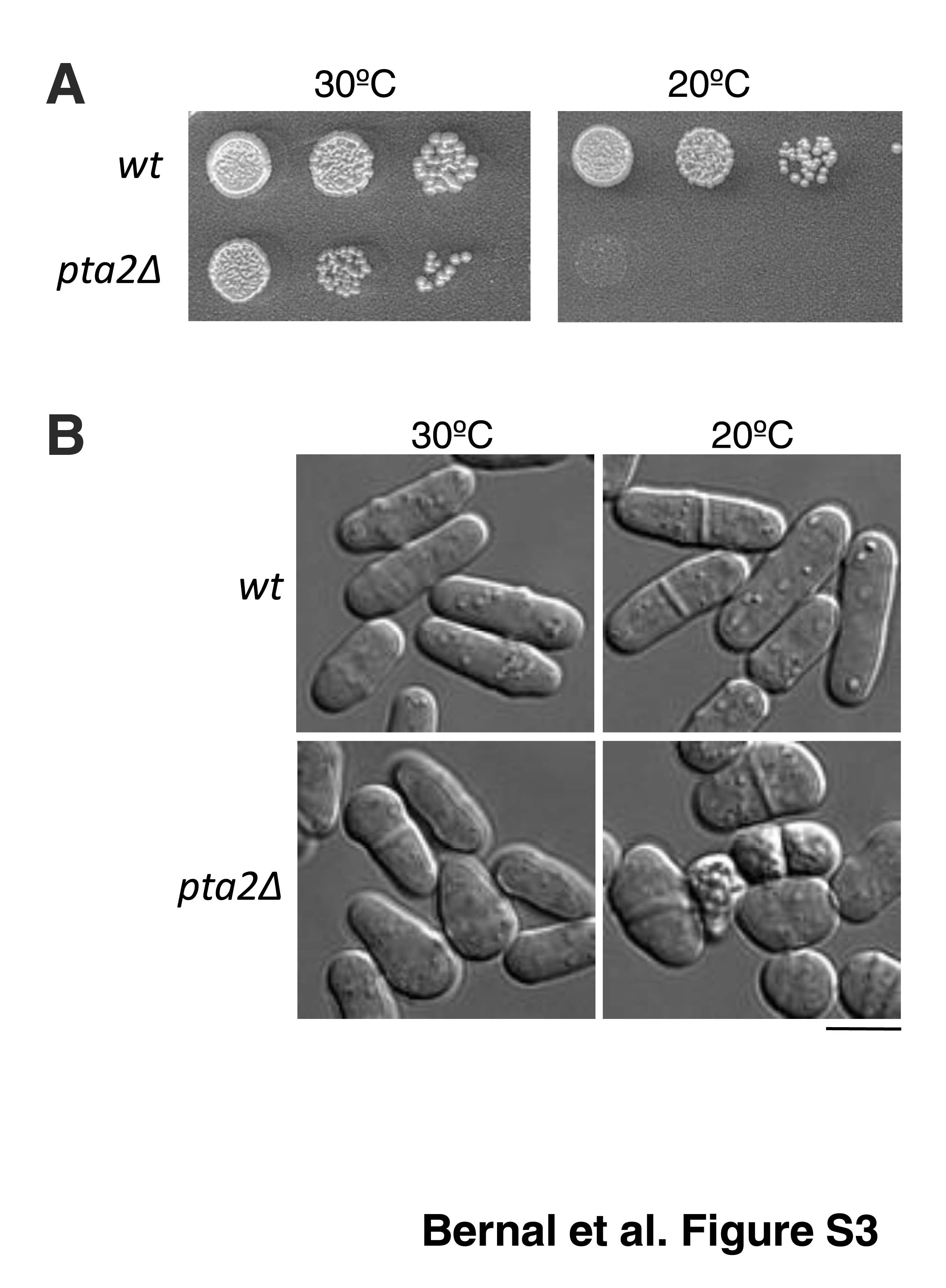

Supplement: Figure S3 — The cold sensitive phenotype of pta2 Δ cells is not suppressed by 1 M sorbitol. A. Growth assay of serial dilutions of wild type and pta2Δ cells at the indicated temperatures in YES agar containing 1 M sorbitol. B. Differential interference contrast (DIC) images of wild type and pta2Δ cells grown at the indicated temperatures. in YES medium containing 1 M sorbitol. Scale bar, 5 µm. (TIF) [file pone.0032823.s003.tif]

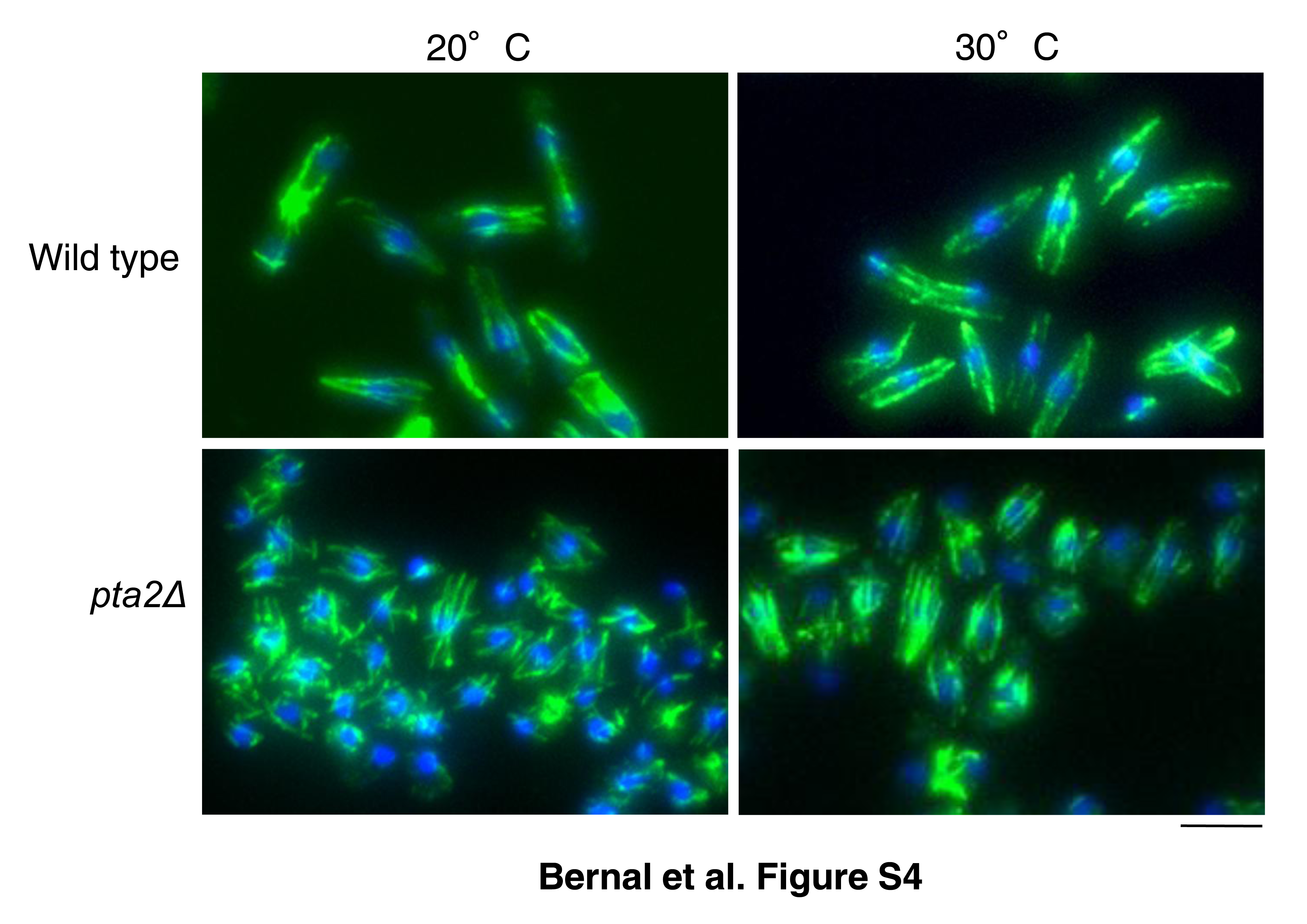

Supplement: Figure S4 — Microtubule cytoskeleton in wild type and pta2 Δ cells. Wild type and pta2Δ cells were grown at either 25°C or 30°C and then fixed with methanol and incubated with anti-tubulin (TAT1) primary antibody and anti-mouse Alexa fluor 488 secondary antibody. Images were taken in multiple focal planes, maximum projections are shown. DAPI staining is shown in bottom panels. Scale bar, 5 µm. (TIF) [file pone.0032823.s004.tif]

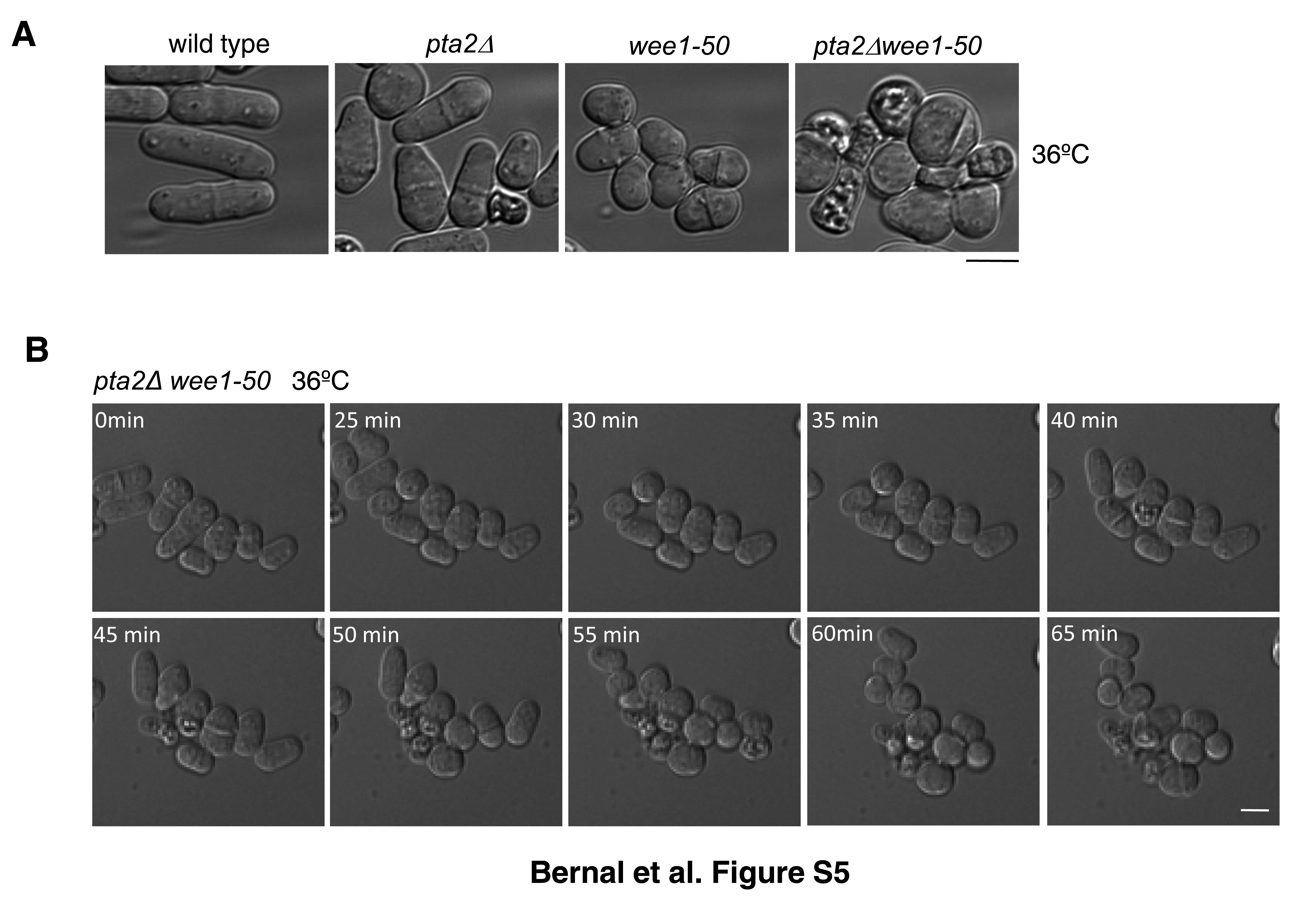

Supplement: Figure S5 — Synthetic lethality of pta2 Δ wee1-50 strain at 36°C. A. DIC images of the indicated strains after 6 hours incubation at 36°C. B. The pta2Δwee1-50 strain was grown at 25°C and then shifted to 36°C (at time 0 hrs). Images were taken every 5 minutes at a constant temperature of 36°C, representative time points are shown. Scale bar, 5 µm. (TIF) [file pone.0032823.s005.tif]
